# Poly[tetra­aqua-di-μ_4_-malonato-barium(II)cadmium(II)]

**DOI:** 10.1107/S1600536810049780

**Published:** 2010-12-04

**Authors:** Ming-Lin Guo, Wen-Jun Gao, Cong-Cong Luo, Long Liu

**Affiliations:** a School of Environment and Chemical Engineering and, Key Laboratory of Hollow Fiber Membrane Materials & Membrane Processes, Tianjin Polytechnic University, Tianjin 300160, People’s Republic of China

## Abstract

In the title complex, [BaCd(C_3_H_2_O_4_)_2_(H_2_O)_4_]_*n*_, the Ba^II^ atoms, located on crystallographic twofold axes, adopt slightly distorted square-anti­prismatic coordination geometries, while the Cd^II^ atoms, which lie on crystallographic centres of symmetry, have a distorted octa­hedral coordination. Each malonate dianion binds two different Cd^II^ atoms and two different Ba^II^ atoms. This connectivity generates alternating layers along [100] in the structure, with one type containing Cd^II^ cations and malonate dianions, while the other is primarily composed of Ba^II^ ions and coordinated water mol­ecules. The water mol­ecules also participate in extensive O—H⋯O hydrogen bonding.

## Related literature

For structural studies on the malonate dianion with its versatile coordination patterns, see: Delgado *et al.* (2004 ▶). For related structures, see Djeghri *et al.* (2005 ▶); Guo & Guo (2006 ▶). 
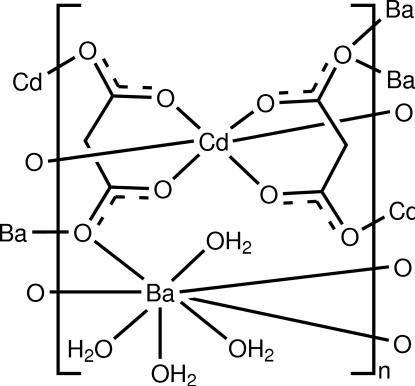


## Experimental

### 

#### Crystal data

[BaCd(C_3_H_2_O_4_)_2_(H_2_O)_4_]
*M* *_r_* = 525.90
Orthorhombic, 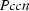

*a* = 18.809 (4) Å
*b* = 6.9224 (14) Å
*c* = 9.6849 (19) Å
*V* = 1261.0 (4) Å^3^
*Z* = 4
Mo *K*α radiation
μ = 4.85 mm^−1^
*T* = 294 K
0.24 × 0.20 × 0.10 mm

#### Data collection

Bruker SMART CCD area-detector diffractometer
Absorption correction: multi-scan (*SADABS*; Sheldrick, 2000 ▶) *T*_min_ = 0.370, *T*_max_ = 0.662
5716 measured reflections
1103 independent reflections
978 reflections with *I* > 2σ(*I*)
*R*_int_ = 0.059

#### Refinement

*R*[*F* ^2^ > 2σ(*F* ^2^)] = 0.060
*wR*(*F* ^2^) = 0.161
*S* = 1.06
1103 reflections
94 parameters
H-atom parameters constrained
Δρ_max_ = 1.94 e Å^−3^
Δρ_min_ = −1.24 e Å^−3^

### 

Data collection: *SMART* (Bruker, 1997 ▶); cell refinement: *SAINT* (Bruker, 1997 ▶); data reduction: *SAINT*; program(s) used to solve structure: *SHELXS97* (Sheldrick, 2008 ▶); program(s) used to refine structure: *SHELXL97* (Sheldrick, 2008 ▶); molecular graphics: *SHELXTL* (Sheldrick, 2008 ▶); software used to prepare material for publication: *SHELXTL*.

## Supplementary Material

Crystal structure: contains datablocks I, global. DOI: 10.1107/S1600536810049780/sj5064sup1.cif

Structure factors: contains datablocks I. DOI: 10.1107/S1600536810049780/sj5064Isup2.hkl

Additional supplementary materials:  crystallographic information; 3D view; checkCIF report

## Figures and Tables

**Table 1:** Selected bond lengths (Å)

Ba1—O4^i^	2.794 (9)
Ba1—O6	2.809 (10)
Ba1—O4	2.854 (9)
Ba1—O5	2.877 (10)
Cd1—O2^ii^	2.227 (10)
Cd1—O3	2.227 (9)
Cd1—O1^iii^	2.364 (8)

Symmetry codes: (i) 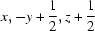; (ii) 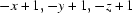; (iii) 
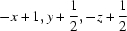
.

**Table 2:** Hydrogen-bond geometry (Å, °)

*D*—H⋯*A*	*D*—H	H⋯*A*	*D*⋯*A*	*D*—H⋯*A*
O6—H6*B*⋯O5^iii^	0.87	2.08	2.893 (14)	157
O6—H6*A*⋯O1^iv^	0.85	1.99	2.781 (13)	156
O5—H5*B*⋯O6^i^	0.87	2.19	2.919 (15)	141
O5—H5*A*⋯O2^ii^	0.84	2.01	2.810 (14)	159

Symmetry codes: (i) 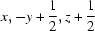; (ii) 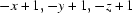; (iii) 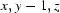; (iv) 
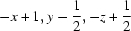
.

**Fig. 1.**
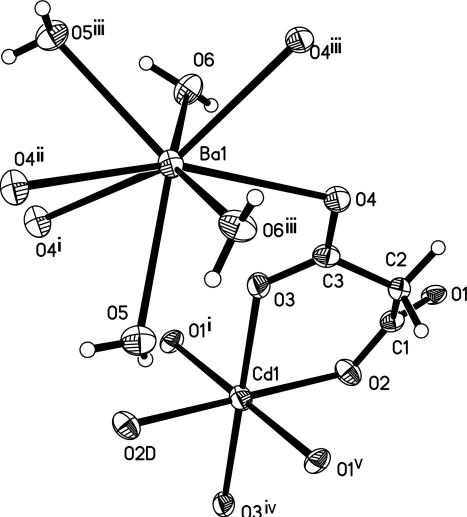
The coordination of the metal ions in (I). Displacement ellipsoids are drawn at the 30% probability level. Symmetry codes (i) x, -y + 1/2, z + 1/2; (ii) -x + 1/2, y, z + 1/2; (iii) -x + 1/2, -y + 1/2, z; (iv) -x + 1, -y + 1, -z + 1; (v) -x + 1, y + 1/2, -z + 1/2.

**Fig. 2.**
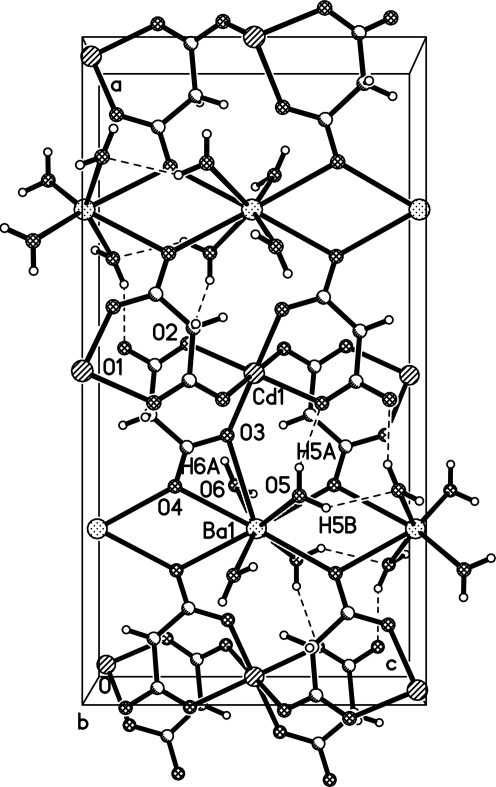
A view, approximately along the b axis, showing the alternation of type 1 and type 2 layers along the a axis.

## supplementary crystallographic information

### Comment

The malonate dianion, with two neighboring carboxylate groups, is a very flexible ligand. Its basic coordination mode is as a chelate *via* two distal carboxylate oxygen atoms to form a six-membered ring and the coordinating ability of the nonchelating oxygen atoms makes the formation of polymeric networks possible (Djeghri *et al.*, 2005; Guo & Guo, 2006). On the other hand, malonate can also coordinate  in monodentate, chelated bidentate and bridging modes to create various molecular architectures (Delgado *et al.*, 2004). Herein, we report the structure of the title heterobimetallic malonate complex, (I). It and the chemically similar complex poly[tetraaqua-di-mu4-malonato-barium(II)zinc(II)] (Guo & Guo, 2006) are isotypic.

The asymmetric unit in the structure of (I) comprises half a Ba^II^ cation, half a Cd^II^ cation, a complete malonate dianion defined by C1—C3/O1—O4 and two independent water molecules involving O5 and O6. Fig. 1 shows a symmetry-expanded view which displays the full coordination of the Ba^2+^ and Cd^2+^ centers. Selected geometric parameters are given in Table 1.

The Ba^2+^ cation, lying on a crystallographic twofold axis, is eight-coordinate, bonded to oxygen atoms of four different malonate groups and four water molecules with Ba—O distances ranging from 2.793 (9) to 2.878 (10) Å. The Ba polyhedra may be described as slightly distorted square antiprisms. They share edges to form chains propagating along *c*.

The Cd^2+^ cations, lie on crystallographic centres of symmetry, and have distorted octahedral coordination, with O2 and O3 of two bidentate malonate anions at the equatorial sites and two O1 atoms from two other malonate anions at the apical sites.

Also evident in Fig. 1 is the variability of the coordination modes of the malonate dianion with monodentate (O1), bidentate chelating (O2 and O3) and bridging (O4) bonding modes all present.

The structure as a whole consists of two distinct types of layer, both parallel to (100) and stacked alternately in the direction of a. The first of these (Fig. 2) is composed entirely of Cd^II^ ions and malonate dianions and occurs at *x* = 0 and 1/2. The other type of layer, type 2, alternating with the first and centred on *x* = 1/4 and 3/4 contains, primarily, the Ba ions and the water molecules. Two forms of connectivity occur within the type 2 layers. First of all O4 atoms on the surfaces of the type 1 layers create chains of edge sharing Ba polyhedra propagating along *c* and at the same time link the two types of layer and complete the three-dimensional connectivity of the structure. The interlayer connectivity is further enhanced by the hydrogen bonds of the form O5—H5A···O2^iv^ and O6—H6A···O1^vi^ given in Table 2.

### Experimental

The title complex was prepared under continuous stirring with successive addition of malonic acid (0.43 g, 4 mmol), cadmium(II) chloride (0.37 g, 2 mmol) and Ba(OH)_2_.8H_2_O (0.63 g, 2 mmol) to distilled water (40 ml) at room temperature. After filtration, slow evaporation over a period of a week at room temperature provided colorless plate-like crystals of (I).

### Refinement

The H atoms of the water molecule were found in difference Fourier maps and during refinement were fixed at an O–H distance of 0.85 Å, and with *U*_iso_(H) = 1.2 *U*_eq_(O). The H atoms of C–H groups were placed geometrically and during refinement were treated using a riding model, with C–H = 0.97 Å, and with *U*_iso_(H) = 1.2 *U*_eq_(C).

### Crystal data

**Table:**

[BaCd(C_3_H_2_O_4_)_2_(H_2_O)_4_]	*F*(000) = 992
*M**_r_* = 525.90	*D*_x_ = 2.770 Mg m^−^^3^
Orthorhombic, *P**c**c**n*	Mo *K*α radiation, λ = 0.71073 Å
Hall symbol: -P 2ab 2ac	Cell parameters from 3576 reflections
*a* = 18.809 (4) Å	θ = 3.1–26.4°
*b* = 6.9224 (14) Å	µ = 4.85 mm^−^^1^
*c* = 9.6849 (19) Å	*T* = 294 K
*V* = 1261.0 (4) Å^3^	Prism, colorless
*Z* = 4	0.24 × 0.20 × 0.10 mm

### Data collection

**Table:**

Bruker SMART CCD area-detector diffractometer	1103 independent reflections
Radiation source: sealed tube	978 reflections with *I* > 2σ(*I*)
graphite	*R*_int_ = 0.059
φ and ω scans	θ_max_ = 25.0°, θ_min_ = 2.2°
Absorption correction: multi-scan (*SADABS*; Sheldrick, 2000)	*h* = −22→10
*T*_min_ = 0.370, *T*_max_ = 0.662	*k* = −8→8
5716 measured reflections	*l* = −10→11

### Refinement

**Table:**

Refinement on *F*^2^	Secondary atom site location: difference Fourier map
Least-squares matrix: full	Hydrogen site location: inferred from neighbouring sites
*R*[*F*^2^ > 2σ(*F*^2^)] = 0.060	H-atom parameters constrained
*wR*(*F*^2^) = 0.161	*w* = 1/[σ^2^(*F*_o_^2^) + (0.0364*P*)^2^ + 63.5907*P*] where *P* = (*F*_o_^2^ + 2*F*_c_^2^)/3
*S* = 1.06	(Δ/σ)_max_ = 0.001
1103 reflections	Δρ_max_ = 1.94 e Å^−^^3^
94 parameters	Δρ_min_ = −1.24 e Å^−^^3^
0 restraints	Extinction correction: *SHELXL97* (Sheldrick, 2008), Fc^*^=kFc[1+0.001xFc^2^λ^3^/sin(2θ)]^-1/4^
Primary atom site location: structure-invariant direct methods	Extinction coefficient: 0.0147 (13)

### Special details

**Table:**

Geometry. All e.s.d.'s (except the e.s.d. in the dihedral angle between two l.s. planes) are estimated using the full covariance matrix. The cell e.s.d.'s are taken into account individually in the estimation of e.s.d.'s in distances, angles and torsion angles; correlations between e.s.d.'s in cell parameters are only used when they are defined by crystal symmetry. An approximate (isotropic) treatment of cell e.s.d.'s is used for estimating e.s.d.'s involving l.s. planes.
Refinement. Refinement of *F*^2^ against ALL reflections. The weighted *R*-factor *wR* and goodness of fit *S* are based on *F*^2^, conventional *R*-factors *R* are based on *F*, with *F* set to zero for negative *F*^2^. The threshold expression of *F*^2^ > σ(*F*^2^) is used only for calculating *R*-factors(gt) *etc*. and is not relevant to the choice of reflections for refinement. *R*-factors based on *F*^2^ are statistically about twice as large as those based on *F*, and *R*- factors based on ALL data will be even larger.

### Fractional atomic coordinates and isotropic or equivalent isotropic displacement parameters (Å^2^)

**Table:**

	*x*	*y*	*z*	*U*_iso_*/*U*_eq_	
Ba1	0.2500	0.2500	0.50669 (11)	0.0320 (5)	
Cd1	0.5000	0.5000	0.5000	0.0320 (6)	
O1	0.5419 (5)	0.2940 (12)	0.0939 (9)	0.030 (2)	
O2	0.5475 (5)	0.4394 (15)	0.2939 (10)	0.035 (2)	
O3	0.4014 (5)	0.3657 (15)	0.4162 (9)	0.034 (2)	
O4	0.3196 (5)	0.3410 (17)	0.2530 (9)	0.035 (2)	
O5	0.3078 (5)	0.5890 (15)	0.6370 (11)	0.041 (3)	
H5B	0.2892	0.6078	0.7177	0.061*	
H5A	0.3517	0.5665	0.6370	0.061*	
O6	0.3163 (5)	−0.1005 (15)	0.4378 (11)	0.041 (2)	
H6A	0.3571	−0.1227	0.4039	0.062*	
H6B	0.3010	−0.1850	0.4966	0.062*	
C1	0.5135 (7)	0.3835 (18)	0.1916 (14)	0.029 (3)	
C2	0.4344 (7)	0.4323 (19)	0.1784 (13)	0.028 (3)	
H2A	0.4307	0.5714	0.1686	0.033*	
H2B	0.4178	0.3763	0.0924	0.033*	
C3	0.3825 (7)	0.3720 (19)	0.2905 (15)	0.030 (3)	

### Atomic displacement parameters (Å^2^)

**Table:**

	*U*^11^	*U*^22^	*U*^33^	*U*^12^	*U*^13^	*U*^23^
Ba1	0.0311 (8)	0.0367 (8)	0.0281 (7)	0.0015 (5)	0.000	0.000
Cd1	0.0341 (9)	0.0357 (9)	0.0261 (8)	−0.0001 (6)	−0.0021 (6)	−0.0004 (6)
O1	0.035 (5)	0.018 (4)	0.037 (5)	−0.003 (4)	0.005 (4)	−0.005 (4)
O2	0.028 (5)	0.045 (6)	0.033 (5)	0.004 (5)	−0.006 (4)	−0.007 (5)
O3	0.031 (5)	0.046 (6)	0.026 (5)	−0.009 (4)	−0.002 (4)	0.003 (4)
O4	0.020 (5)	0.052 (6)	0.032 (5)	−0.010 (5)	−0.001 (4)	0.001 (4)
O5	0.030 (5)	0.041 (6)	0.052 (6)	0.001 (5)	−0.006 (5)	−0.001 (5)
O6	0.031 (5)	0.044 (6)	0.049 (6)	0.004 (5)	0.011 (5)	0.006 (5)
C1	0.031 (7)	0.021 (6)	0.034 (7)	0.001 (5)	0.000 (6)	0.001 (5)
C2	0.029 (7)	0.029 (7)	0.025 (6)	−0.003 (6)	0.001 (6)	0.000 (5)
C3	0.032 (7)	0.023 (7)	0.036 (7)	0.001 (6)	−0.002 (6)	−0.003 (6)

### Geometric parameters (Å, °)

**Table:**

Ba1—O4^i^	2.794 (9)	Cd1—O1^v^	2.364 (8)
Ba1—O4^ii^	2.794 (9)	Cd1—O1^i^	2.364 (8)
Ba1—O6^iii^	2.809 (10)	O1—C1	1.252 (16)
Ba1—O6	2.809 (10)	O1—Cd1^vi^	2.364 (8)
Ba1—O4^iii^	2.854 (9)	O2—C1	1.241 (16)
Ba1—O4	2.854 (9)	O3—C3	1.269 (17)
Ba1—O5	2.877 (10)	O4—C3	1.255 (16)
Ba1—O5^iii^	2.877 (10)	O4—Ba1^vii^	2.794 (9)
Ba1—O3^iii^	3.086 (9)	O5—H5B	0.8658
Ba1—O3	3.086 (9)	O5—H5A	0.8410
Ba1—C3^iii^	3.363 (14)	O6—H6A	0.8479
Ba1—C3	3.363 (14)	O6—H6B	0.8659
Cd1—O2^iv^	2.227 (10)	C1—C2	1.531 (18)
Cd1—O2	2.227 (10)	C2—C3	1.518 (19)
Cd1—O3^iv^	2.227 (9)	C2—H2A	0.9700
Cd1—O3	2.227 (9)	C2—H2B	0.9700
			
O4^i^—Ba1—O4^ii^	62.7 (4)	O4^i^—Ba1—C3	103.9 (3)
O4^i^—Ba1—O6^iii^	127.3 (3)	O4^ii^—Ba1—C3	145.4 (3)
O4^ii^—Ba1—O6^iii^	78.5 (3)	O6^iii^—Ba1—C3	88.0 (3)
O4^i^—Ba1—O6	78.5 (3)	O6—Ba1—C3	74.9 (3)
O4^ii^—Ba1—O6	127.3 (3)	O4^iii^—Ba1—C3	81.9 (3)
O6^iii^—Ba1—O6	152.5 (4)	O4—Ba1—C3	21.3 (3)
O4^i^—Ba1—O4^iii^	154.2 (5)	O5—Ba1—C3	77.8 (3)
O4^ii^—Ba1—O4^iii^	124.7 (4)	O5^iii^—Ba1—C3	139.3 (3)
O6^iii^—Ba1—O4^iii^	77.4 (3)	O3^iii^—Ba1—C3	124.9 (3)
O6—Ba1—O4^iii^	79.0 (3)	O3—Ba1—C3	22.2 (3)
O4^i^—Ba1—O4	124.7 (4)	C3^iii^—Ba1—C3	103.0 (5)
O4^ii^—Ba1—O4	154.2 (5)	O2^iv^—Cd1—O2	180.000 (1)
O6^iii^—Ba1—O4	79.0 (3)	O2^iv^—Cd1—O3^iv^	85.9 (3)
O6—Ba1—O4	77.4 (3)	O2—Cd1—O3^iv^	94.1 (3)
O4^iii^—Ba1—O4	61.2 (4)	O2^iv^—Cd1—O3	94.1 (3)
O4^i^—Ba1—O5	68.4 (3)	O2—Cd1—O3	85.9 (3)
O4^ii^—Ba1—O5	67.6 (3)	O3^iv^—Cd1—O3	180.0 (3)
O6^iii^—Ba1—O5	64.4 (3)	O2^iv^—Cd1—O1^v^	92.8 (3)
O6—Ba1—O5	129.8 (3)	O2—Cd1—O1^v^	87.2 (3)
O4^iii^—Ba1—O5	136.9 (3)	O3^iv^—Cd1—O1^v^	93.3 (3)
O4—Ba1—O5	91.4 (3)	O3—Cd1—O1^v^	86.7 (3)
O4^i^—Ba1—O5^iii^	67.6 (3)	O2^iv^—Cd1—O1^i^	87.2 (3)
O4^ii^—Ba1—O5^iii^	68.4 (3)	O2—Cd1—O1^i^	92.8 (3)
O6^iii^—Ba1—O5^iii^	129.8 (3)	O3^iv^—Cd1—O1^i^	86.7 (3)
O6—Ba1—O5^iii^	64.4 (3)	O3—Cd1—O1^i^	93.3 (3)
O4^iii^—Ba1—O5^iii^	91.4 (3)	O1^v^—Cd1—O1^i^	180.0 (4)
O4—Ba1—O5^iii^	136.9 (3)	C1—O1—Cd1^vi^	125.1 (8)
O5—Ba1—O5^iii^	128.0 (4)	C1—O2—Cd1	124.6 (9)
O4^i^—Ba1—O3^iii^	128.1 (3)	C3—O3—Cd1	124.7 (9)
O4^ii^—Ba1—O3^iii^	82.4 (3)	C3—O3—Ba1	91.3 (8)
O6^iii^—Ba1—O3^iii^	75.3 (3)	Cd1—O3—Ba1	140.6 (4)
O6—Ba1—O3^iii^	96.8 (3)	C3—O4—Ba1^vii^	136.6 (9)
O4^iii^—Ba1—O3^iii^	43.5 (3)	C3—O4—Ba1	102.8 (8)
O4—Ba1—O3^iii^	103.7 (3)	Ba1^vii^—O4—Ba1	118.0 (3)
O5—Ba1—O3^iii^	133.2 (3)	Ba1—O5—H5B	111.5
O5^iii^—Ba1—O3^iii^	64.2 (3)	Ba1—O5—H5A	102.7
O4^i^—Ba1—O3	82.4 (3)	H5B—O5—H5A	115.1
O4^ii^—Ba1—O3	128.1 (3)	Ba1—O6—H6A	130.6
O6^iii^—Ba1—O3	96.8 (3)	Ba1—O6—H6B	106.2
O6—Ba1—O3	75.3 (3)	H6A—O6—H6B	115.7
O4^iii^—Ba1—O3	103.7 (3)	O2—C1—O1	122.6 (12)
O4—Ba1—O3	43.5 (3)	O2—C1—C2	119.9 (12)
O5—Ba1—O3	64.2 (3)	O1—C1—C2	117.4 (12)
O5^iii^—Ba1—O3	133.2 (3)	C3—C2—C1	120.2 (11)
O3^iii^—Ba1—O3	147.0 (3)	C3—C2—H2A	107.3
O4^i^—Ba1—C3^iii^	145.3 (3)	C1—C2—H2A	107.3
O4^ii^—Ba1—C3^iii^	103.9 (3)	C3—C2—H2B	107.3
O6^iii^—Ba1—C3^iii^	74.9 (3)	C1—C2—H2B	107.3
O6—Ba1—C3^iii^	88.0 (3)	H2A—C2—H2B	106.9
O4^iii^—Ba1—C3^iii^	21.3 (3)	O4—C3—O3	122.4 (13)
O4—Ba1—C3^iii^	81.9 (3)	O4—C3—C2	116.4 (12)
O5—Ba1—C3^iii^	139.3 (3)	O3—C3—C2	121.0 (12)
O5^iii^—Ba1—C3^iii^	77.8 (3)	O4—C3—Ba1	55.9 (7)
O3^iii^—Ba1—C3^iii^	22.2 (3)	O3—C3—Ba1	66.6 (7)
O3—Ba1—C3^iii^	124.9 (3)	C2—C3—Ba1	172.1 (9)
			
O3^iv^—Cd1—O2—C1	−170.4 (11)	O6—Ba1—O4—Ba1^vii^	84.2 (4)
O3—Cd1—O2—C1	9.6 (11)	O4^iii^—Ba1—O4—Ba1^vii^	0.0
O1^v^—Cd1—O2—C1	−77.2 (11)	O5—Ba1—O4—Ba1^vii^	−145.1 (4)
O1^i^—Cd1—O2—C1	102.8 (11)	O5^iii^—Ba1—O4—Ba1^vii^	56.8 (6)
O2^iv^—Cd1—O3—C3	148.6 (11)	O3^iii^—Ba1—O4—Ba1^vii^	−9.8 (5)
O2—Cd1—O3—C3	−31.4 (11)	O3—Ba1—O4—Ba1^vii^	166.0 (7)
O1^v^—Cd1—O3—C3	56.0 (11)	C3^iii^—Ba1—O4—Ba1^vii^	−5.5 (4)
O1^i^—Cd1—O3—C3	−124.0 (11)	C3—Ba1—O4—Ba1^vii^	164.9 (12)
O2^iv^—Cd1—O3—Ba1	−3.7 (7)	Cd1—O2—C1—O1	−156.7 (9)
O2—Cd1—O3—Ba1	176.3 (7)	Cd1—O2—C1—C2	26.4 (17)
O1^v^—Cd1—O3—Ba1	−96.3 (6)	Cd1^vi^—O1—C1—O2	129.4 (11)
O1^i^—Cd1—O3—Ba1	83.7 (6)	Cd1^vi^—O1—C1—C2	−53.6 (14)
O4^i^—Ba1—O3—C3	166.0 (8)	O2—C1—C2—C3	−57.2 (17)
O4^ii^—Ba1—O3—C3	−147.8 (8)	O1—C1—C2—C3	125.7 (13)
O6^iii^—Ba1—O3—C3	−67.2 (8)	Ba1^vii^—O4—C3—O3	−162.7 (10)
O6—Ba1—O3—C3	86.0 (8)	Ba1—O4—C3—O3	−2.2 (15)
O4^iii^—Ba1—O3—C3	11.5 (8)	Ba1^vii^—O4—C3—C2	22 (2)
O4—Ba1—O3—C3	−1.1 (7)	Ba1—O4—C3—C2	−177.9 (9)
O5—Ba1—O3—C3	−124.4 (9)	Ba1^vii^—O4—C3—Ba1	−160.5 (15)
O5^iii^—Ba1—O3—C3	116.7 (8)	Cd1—O3—C3—O4	−160.9 (10)
O3^iii^—Ba1—O3—C3	6.3 (7)	Ba1—O3—C3—O4	2.0 (14)
C3^iii^—Ba1—O3—C3	9.2 (11)	Cd1—O3—C3—C2	14.7 (18)
O4^i^—Ba1—O3—Cd1	−36.5 (7)	Ba1—O3—C3—C2	177.5 (11)
O4^ii^—Ba1—O3—Cd1	9.7 (8)	Cd1—O3—C3—Ba1	−162.9 (10)
O6^iii^—Ba1—O3—Cd1	90.4 (7)	C1—C2—C3—O4	−151.1 (13)
O6—Ba1—O3—Cd1	−116.5 (7)	C1—C2—C3—O3	33.1 (19)
O4^iii^—Ba1—O3—Cd1	169.0 (6)	O4^i^—Ba1—C3—O4	167.7 (7)
O4—Ba1—O3—Cd1	156.5 (9)	O4^ii^—Ba1—C3—O4	−130.5 (10)
O5—Ba1—O3—Cd1	33.2 (6)	O6^iii^—Ba1—C3—O4	−64.3 (9)
O5^iii^—Ba1—O3—Cd1	−85.7 (7)	O6—Ba1—C3—O4	94.0 (9)
O3^iii^—Ba1—O3—Cd1	163.8 (7)	O4^iii^—Ba1—C3—O4	13.3 (10)
C3^iii^—Ba1—O3—Cd1	166.7 (6)	O5—Ba1—C3—O4	−128.5 (9)
C3—Ba1—O3—Cd1	157.5 (13)	O5^iii^—Ba1—C3—O4	96.0 (9)
O4^i^—Ba1—O4—C3	−14.5 (8)	O3^iii^—Ba1—C3—O4	6.2 (10)
O4^ii^—Ba1—O4—C3	84.2 (9)	O3—Ba1—C3—O4	−178.0 (14)
O6^iii^—Ba1—O4—C3	113.5 (9)	C3^iii^—Ba1—C3—O4	9.7 (8)
O6—Ba1—O4—C3	−80.7 (9)	O4^i^—Ba1—C3—O3	−14.3 (8)
O4^iii^—Ba1—O4—C3	−164.9 (12)	O4^ii^—Ba1—C3—O3	47.5 (10)
O5—Ba1—O4—C3	49.9 (9)	O6^iii^—Ba1—C3—O3	113.7 (8)
O5^iii^—Ba1—O4—C3	−108.1 (9)	O6—Ba1—C3—O3	−88.0 (8)
O3^iii^—Ba1—O4—C3	−174.8 (9)	O4^iii^—Ba1—C3—O3	−168.7 (8)
O3—Ba1—O4—C3	1.1 (8)	O4—Ba1—C3—O3	178.0 (14)
C3^iii^—Ba1—O4—C3	−170.4 (8)	O5—Ba1—C3—O3	49.5 (8)
O4^i^—Ba1—O4—Ba1^vii^	150.4 (5)	O5^iii^—Ba1—C3—O3	−86.0 (9)
O4^ii^—Ba1—O4—Ba1^vii^	−110.9 (6)	O3^iii^—Ba1—C3—O3	−175.8 (5)
O6^iii^—Ba1—O4—Ba1^vii^	−81.6 (4)	C3^iii^—Ba1—C3—O3	−172.3 (9)

Symmetry codes: (i) *x*, −*y*+1/2, *z*+1/2; (ii) −*x*+1/2, *y*, *z*+1/2; (iii) −*x*+1/2, −*y*+1/2, *z*; (iv) −*x*+1, −*y*+1, −*z*+1; (v) −*x*+1, *y*+1/2, −*z*+1/2; (vi) −*x*+1, *y*−1/2, −*z*+1/2; (vii) −*x*+1/2, *y*, *z*−1/2.

### Hydrogen-bond geometry (Å, °)

**Table:**

*D*—H···*A*	*D*—H	H···*A*	*D*···*A*	*D*—H···*A*
O6—H6B···O5^viii^	0.87	2.08	2.893 (14)	157.
O6—H6A···O1^vi^	0.85	1.99	2.781 (13)	156.
O5—H5B···O6^i^	0.87	2.19	2.919 (15)	141.
O5—H5A···O2^iv^	0.84	2.01	2.810 (14)	159.

Symmetry codes: (viii) *x*, *y*−1, *z*; (vi) −*x*+1, *y*−1/2, −*z*+1/2; (i) *x*, −*y*+1/2, *z*+1/2; (iv) −*x*+1, −*y*+1, −*z*+1.
